# Unilateral chewing of foods. Analysis of energy balance and peak power of the mandibular elevator muscles

**DOI:** 10.3389/fbioe.2025.1559555

**Published:** 2025-04-01

**Authors:** Przemysław Stróżyk, Jacek Bałchanowski

**Affiliations:** ^1^ Department of Mechanics, Materials and Biomedical Engineering, Faculty of Mechanical Engineering, Wroclaw University of Science and Technology, Wroclaw, Poland; ^2^ Department of Fundamentals of Machine Design and Mechatronic Systems, Faculty of Mechanical Engineering, Wrocław University of Science and Technology, Wrocław, Poland

**Keywords:** mastication, mandibular elevator muscles, numerical simulation, muscle energy, peak muscle power

## Abstract

**Introduction:** The paper presents the results concerning the energy (work) and peak power generated by the elevator muscles of the mandible (the masseter, medial pterygoid, and temporalis muscles) during unilateral chewing of selected food products in vitro. Since the act of chewing is a very complex issue in the biomechanics of the masticatory system, the research and analysis of the obtained results were therefore limited to the first cycle.

**Methods:** Determination of the peak energy and power of the muscles required: (1) preparation of food patterns, in the form of the function *F = f*(Δ*h*)** (force (*F*) vs displacement (Δ*h*)), based on experimental studies and (2) conducting numerical simulations using a 3D kinematic-dynamic model of the human masticatory system.

**Results and Discussion:** Based on the results, the peak energy and power of the muscles were determined based on food patterns. A comparative analysis was also performed to evaluate the energy and peak power generated by the aforementioned muscles during symmetrical incisal biting vs unilateral chewing of the same food products. The results indicate that (1) food height and texture significantly affect muscle energy and (2) the masticatory and medial pterygoid muscles generate more incredible energy and peak power on the working side than on the non working side, while the opposite was observed for the temporalis muscle and (3) comparative analysis showed that food position on the dental arch has a more significant effect on muscle peak power for foods with high texture heterogeneity than for foods with low texture heterogeneity.

## 1 Introduction

Energy (work) produced by muscles and their power are critical parameters associated with the functioning of organisms, enabling movement, postural stabilization, and interaction with the environment. In the context of humans, understanding the mechanisms governing muscle energy and power is crucial not only for biomechanics but also for sports medicine, rehabilitation, and the design of biomechanical systems. Generally, analyzing the energy (work) and power generated by muscles can provide valuable insights into humans' evolution and motor adaptations in various environments.

Determining muscle work, the amount of energy expended by muscles during contraction is a complex process involving both mechanical and biochemical aspects. Muscles generate force, which translates into motion by applying moments of force to joints, a process can be modelled using dynamics equations. Muscle power can be determined based on knowledge of (1) muscle force, muscle contraction, and contraction time or (2) muscle force and contraction velocity.

Given this information, it is evident that the key parameter related to studies on muscle energy and power is muscle force. In general, the fundamental method for determining muscle forces relies on (1) analyzing motion dynamics and (2) biomechanical modelling, in which muscle forces are calculated based on balancing the moments of forces generated by muscles with external moments acting on selected body segments. Furthermore, studies (Kjær, 2004; Narici and Maganaris, 2006) indicate that muscles adapt to varying external loads through muscle fibres’ structure and mechanical properties changes.

In the case of the muscles of the human masticatory system, it has been shown that the adaptation of the muscular system is primarily associated with the mechanical properties of food (Agrawal et al., 1998; Hiiemae et al., 1996; Koolstra, 2002; Mathevon et al., 1995; Shimada et al., 2012), which play a significant role in the act of chewing. Additionally, studies (Mioche et al., 1999; Stróżyk and Bałchanowski, 2018, Stróżyk and Bałchanowski, 2023) indicate that food patterns (typical mechanical characteristics in the form of force vs. displacement) impose individual muscle activity patterns (muscle force vs. muscle contraction), which must adapt to various functional requirements (Fitts et al., 1991) during the act of chewing.

The muscles playing a key role in the mechanical processing of food are the muscles of mastication, which enable its fragmentation and mixing with saliva, ultimately preparing the bolus of food. From a mechanical perspective, this process requires the coordinated activity of muscles, especially the masseter, temporalis, medial, lateral pterygoid, and suprahyoid and infrahyoid muscles.

Determining muscle forces in the masticatory muscles can be achieved using several methods, each with advantages and limitations. The most commonly used methods include (1) electromyography (*EMG*) (Ferrario and Sforza, 1996; Itoh et al., 1997; Mioche et al., 1999; Murray et al., 1999; Pruim et al., 1980; Ferrario et al., 2006; Castroflorio et al., 2008) and (2) biomechanical modeling, primarily based on numerical models (Stróżyk and Bałchanowski, 2016; Stróżyk and Bałchanowski, 2018; Stróżyk and Bałchanowski, 2023; Pachnicz and Stróżyk, 2021; Griffin et al., 1998; Wang et al., 2023; Pinheiro et al., 2021; Antic et al., 2016; Ackland et al., 2015).

Many publications over the past 30 years suggest that *EMG* measurements are most frequently conducted for the masseter and temporalis muscles, as these measurements do not present significant difficulties (Blanksma and van Eijden, 1995; Mioche et al., 1999; Plesh et al., 1996; Roark et al., 2003). In contrast, measuring the lateral and medial pterygoid muscles is challenging due to the intraoral placement of electrodes (Koole et al., 1990; Murray et al., 1999; Wood et al., 1986), which may interfere with the natural chewing pattern of food.

In the context of muscle energy and power, electromyography does not allow the determination of a fundamental parameter such as muscle contraction. Therefore, the data obtained from *EMG* measurements will make determining muscle energy and power impossible. However, an important advantage of *EMG* is that measurements can be made *in vivo*.

An analysis of the act of chewing in terms of muscle energy and power reveals that a solution enabling the simultaneous determination of parameters needed to calculate these values is biomechanical modelling. This approach, however, requires the development of advanced numerical models utilizing principles of solid mechanics or deformable body mechanics. In the first case, muscles are modeled using vectors (Pachnicz and Stróżyk, 2021; Stróżyk and Bałchanowski, 2016, Stróżyk and Bałchanowski, 2018, Stróżyk and Bałchanowski, 2023; Gross et al., 2001; Gröning et al., 2012; Langenbach and Hannam, 1999), while in the second case, muscle models are based on Hill’s model or volumetric models (Zajac, 1989; Winters, 1990; Sagl et al., 2019; De Zee et al., 2007; Röhrle and Pullan, 2007).

Literature analysis indicates that the kinematic-dynamic model is an optimal model enabling the simultaneous determination of muscle force, contraction, and contraction velocity and, thus, muscle energy and power as a function of food texture. However, its application requires preparing appropriate boundary conditions based on the dynamic characteristics of food (Stróżyk and Bałchanowski, 2018, Stróżyk and Bałchanowski, 2023) and incisal point paths (trajectory) (Stróżyk and Bałchanowski, 2023; Bhatka et al., 2004; Buschang et al., 2007; Nishigawa et al., 1997; Piancino et al., 2012; Slavicek, 2010).

An interesting solution involves integrating computational models (e.g., kinematic-dynamic models) with *EMG* data and imaging techniques (e.g., magnetic resonance imaging (*MRI*) and computed tomography (*CT*)), allowing for more precise determination of muscle forces and analysis of various factors influencing their values, such as changes in maxilla and mandible geometry or malocclusion (Tanne et al., 1995; van Eijden et al., 2003).

The primary aim of this study was to determine the energy and peak power generated by the elevator muscles of the mandible (the masseter, medial pterygoid, and temporalis muscles) for the working and non-working sides during unilateral chewing as a function of selected foods.

Numerical simulations were based on inverse kinematic and dynamic analysis (Lenton et al., 2018; Neptune and van den Bogert, 1998), where the variables were food patterns (classical characteristics in the form of force vs. displacement functions) and kinematic inputs in the form of incisal point paths (trajectory) corresponding to selected foods (Stróżyk and Bałchanowski, 2016, Stróżyk and Bałchanowski, 2018, Stróżyk and Bałchanowski, 2023).

For this study, a proprietary kinematic-dynamic model (Stróżyk and Bałchanowski, 2016, Stróżyk and Bałchanowski, 2018, Stróżyk and Bałchanowski 2023) was developed and utilized to conduct simulations that determined muscle energy and power depending on the food. Additionally, a comparative analysis was conducted to compare the energy and peak power generated by the elevator muscles of the mandible during unilateral chewing (Stróżyk and Bałchanowski, 2020) and the symmetrical incisal biting of the same foods.

During the act of chewing, the kinematic-dynamic parameters of the masticatory system adapt to changing boundary conditions related to variations in (1) the mechanical properties and geometric dimensions of food and (2) the food’s position on the dental arches. Consequently, the study and analysis of results were limited to the first cycle for both unilateral chewing and symmetrical incisal biting.

Mechanical analysis of the masticatory system indicates that it is an advanced biomechanical system used for dynamic food processing (Stokes et al., 2013). Thus, determining the energy (work) and peak power produced by the elevator muscles of the mandible may be helpful for (1) understanding the functioning of the masticatory system and (2) diagnosing and treating disorders of the stomatognathic system. Furthermore, the presented results can also be utilized in designing mechatronic systems inspired by the mechanical functioning of the masticatory system.

## 2 Material and methods

Determination of energy (work) and peak power for the elevator muscles of the mandible (masseter muscle (M), medial pterygoid muscle (*P*) and temporalis muscle (*T*)) during unilateral mastication required (1): preparation of a numerical model of the human masticatory system (Stróżyk and Balchanowski, 2023), (2) development of external loading patterns.

The computational model of the human masticatory system consisted of two members, i.e., a stationary skull and a movable mandible. The external load of the model was the characteristics of the food (*i*) in the form of the function *F*_*i*_ = *f*(Δ*h*_*i*_) - force (*F*_*i*_) vs displacement (Δ*h*_*i*_), determined from experimental studies of unilateral chewing of food. On the other hand, the initial positions of the model were determined based on the prepared chewing loops individually for each food (Stróżyk and Balchanowski, 2023). The computational model developed based on the aforementioned guidelines was used to conduct simulation studies of unilateral chewing of selected foods to determine the kinematic and dynamic parameters necessary to determine the energy and peak power of the muscles.

The present work continues the article on unilateral biting (Stróżyk and Balchanowski, 2023). Therefore, selected aspects of food testing and numerical simulations are described in an abbreviated form compared to the article.

### 2.1 Determination of model load and food characteristics

**FIGURE 1:**
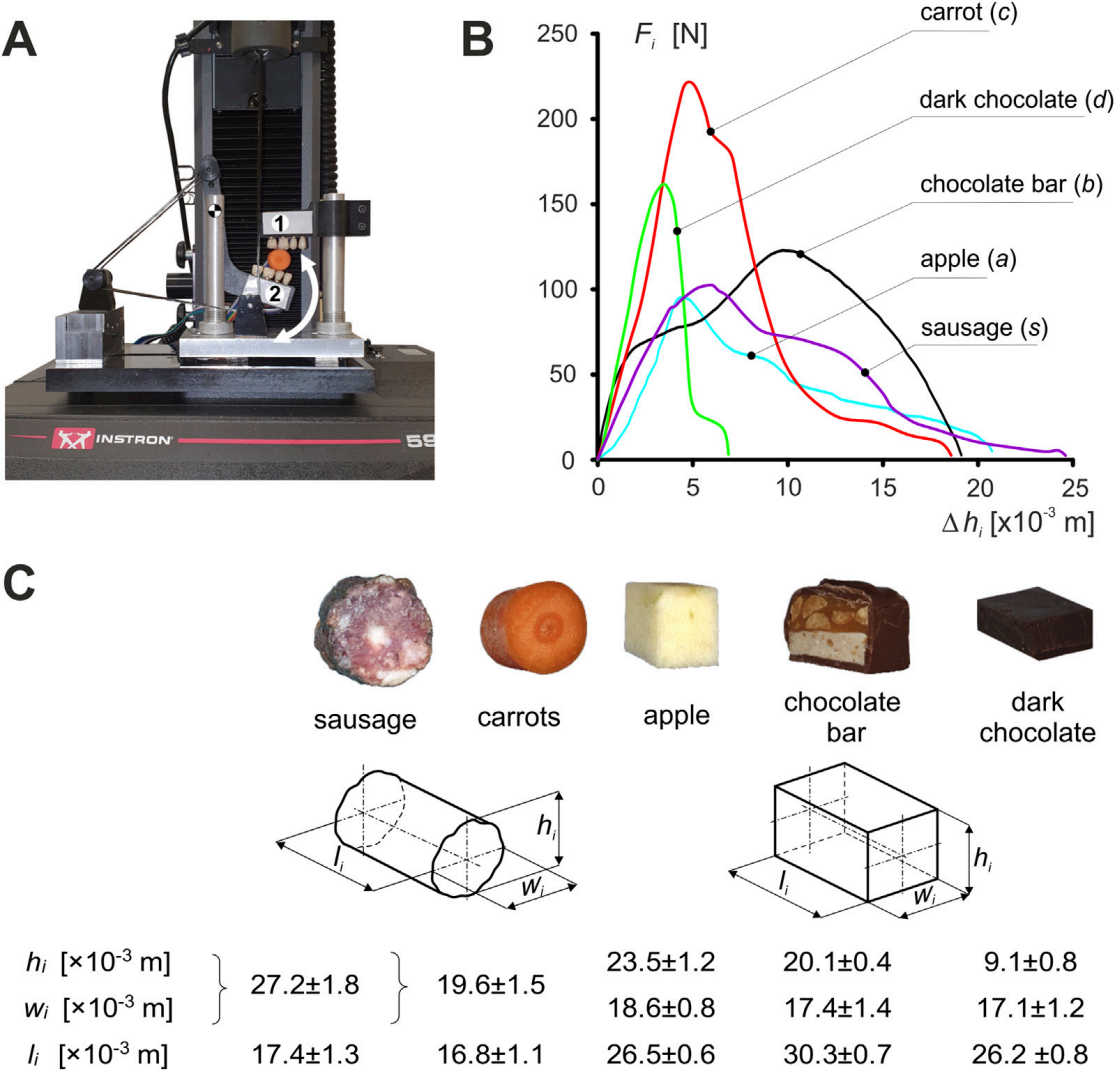
Experimental studies: **(A)** unilateral chewing simulator - one upper grip (maxilla), two lower grip (mandible)) [developed based on a patent application - Stróżyk, 2021], **(B)** characteristics of foods (*i*) and **(C)** mean values of the dimensions of food samples ±SD. (*i* = *c*, *s*, *a*, *b*, *d*).

The general algorithm for determining the characteristics of foods corresponding to unilateral chewing, as well as incisal biting, is described in detail in Stróżyk and Bałchanowski (2016), Stróżyk and Bałchanowski (2018), and Stróżyk and Bałchanowski (2023); Stróżyk et al. 2018. An in-house designed test rig (Figure 1A) (developed based on a patent application (Stróżyk, 2021) was used in the experimental study to determine the characteristics of foods corresponding to the first cycle of unilateral chewing.

The measuring system consisted of two grips (holding grips), i.e., the upper (maxilla) and lower (mandible), imitating fragments of dental arches. To make the tests similar, mechanically, to natural unilateral chewing, acrylic dental prostheses (premolars and molars) were attached to each holder. During testing, the stand was mounted on an *Instron* 5944 test machine. All foods were tested at the same chewing velocity of *V* = 0.02 m/s (Stróżyk and Balchanowski, 2023).

Five foods were used in the study (*i* = *c* (carrot), *a* (apple), *d* (dark chocolate), *b* (chocolate bar), *s* (sausage)) (Stróżyk and Balchanowski, 2016, Stróżyk and Bałchanowski, 2018, and Stróżyk and Bałchanowski, 2023). The samples in terms of dimensions (height (*h*_*i*_), width (*w*_*i*_) and length (*l*_*i*_)) were similar to a typical bite of food, while the shape was product-dependent (Figure 1C).

Based on the results, the characteristics (patterns) of the selected foods were determined in the form of the classical function *F*_*i*_ = *f*(Δ*h*_*i*_) (Figure 1B).

### 2.2 Numerical model of the human masticatory system

**FIGURE 2:**
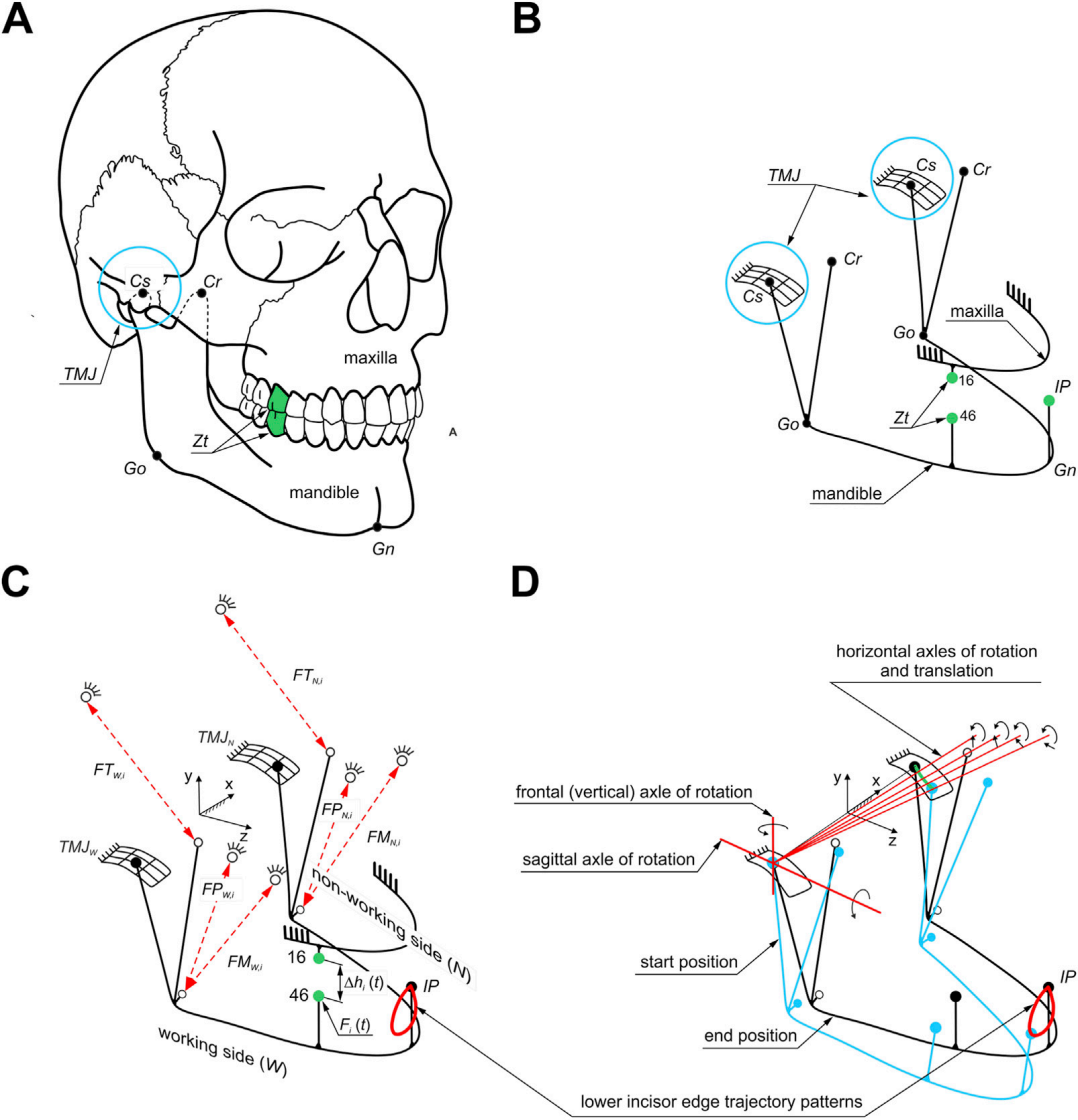
Model of the masticatory system: **(A)** location of selected anthropometric points (*Go*, *Gn*, *Cr*, *Cs*) on the mandible and a pair of corresponding molars (*Zt*), **(B)** geometric model, **(C)** computational model of unilateral chewing. *Symbols (1): anthropometric points: Go - location of the junction of the mandibular branch and mandibular body, Gn - the lowest point on the mandible, Cr - the highest point on the beak process of the mandible, Cs - the highest point on the mandibular condyle (2), points corresponding to the selected pair of molars (Zt) (teeth 46-16) (3), muscle forces: masseter muscle (F*_*MW,i*_ *, F*_*MN,i*_ *), medial pterygoid muscle (F*_*PW,i*_ *, F*_*PN,i*_ *) and temporalis muscle (F*_*TW,i*_ *, F*_*TN,i*_ *) and (4) incisal point (IP) including the masticatory loop*, and **(D)** kinematics of the masticatory system during unilateral biting.

Determination of the energy (work) and peak power of the elevator muscle of the mandible required a dynamic analysis of unilateral chewing of selected foods. For this purpose, a computational model was prepared (Stróżyk and Balchanowski, 2023), the geometry of which was prepared based on anatomical models (skull 8,500 and mandible 8,596) from Synbone (*SYNBONE AG*, *Tardisstrasse 199*, *7*,*205 Zizers*, *Switzerland*). Then, based on the geometric model (Figure 2A), the anthropometric points of the mandible (Phuntsho et al., 2018; Saini et al., 2021) were introduced based on which its model was prepared as a rigid solid (Figure 2B). The maxilla, on the other hand, was modelled in the form of stationary support correlated with corresponding points on the mandible, which in effect made it possible to model a pair of corresponding teeth between which an occlusal force (*F*_*i*_) acts as a function *F*_*i*_ = *f*(Δ*h*_*i*_). The force was applied at a point on the occlusal surface of the first molar (Stróżyk and Bałchanowski, 2023). The muscles were modelled using linear kinematic forcing, which allows the mandible to move relative to the maxilla during chewing (Stróżyk and Balchanowski, 2023). A schematic of the geometric and computational model is shown in Figure 2.

The mandibular model at baseline is fixed at the *TMJ* and the initial attachment points of the masseter muscle, medial pterygoid muscle and temporalis muscle (Figure 2C).

In addition, the numerical simulation assumed that the mandibular condyle on the working side would have a fixed centre of rotation through which the instantaneous axes of rotation would pass. In contrast, the condyle on the non-working side would be able to rotate and translate (Figure 2D).

Based on the developed chewing loops (Stróżyk and Balchanowski, 2023), there are contact kinematic pairs with five degrees of freedom (three rotations and two displacements) in the *TMJ* on the working (*W*) and non-working (*N*) sides. The mandible’s movement model relative to the skull during unilateral chewing is based on the function describing the chewing loops specified for foods. The functions of the chewing loops describing the movement of the *IP* point (Figure 2C) on the mandibular incisor during unilateral chewing of food allowed us to determine the functions of changes in the elongation of individual muscles during chewing (Figure 2; Stróżyk and Balchanowski, 2023). The proposed way of fixing the model allowed for the simulation of the complex movement of the lower incisors during chewing (Figure 2). A detailed description of the model can be found in Stróżyk and Balchanowski (2023).

## 3 Results

The kinematic and dynamic parameters necessary to determine the energy (work) and peak power of the elevator muscles of mandible were determined in two stages: in the first stage, experimental studies were carried out to determine food patterns (Figure 1), while in the second stage, numerical simulations of unilateral chewing were carried out, based on which muscle forces, muscle contractions and muscle contraction time were determined on the working (*W*) and non-working (*N*) sides, respectively, depending on the food pattern.

Since the calculations and analyses were comparative, the simulation assumed an identical chewing velocity with the molars, i.e., *V* = 0.02 m/s (Stróżyk and Balchanowski, 2023). The energy (work) required to bite (*E*_*Xi*_) the selected foods was determined from the characteristics of the foods (Figure 1) based on Equation 1.

$$E_{Xi}=\int_{0}^{h_{\max,i}}F_{i}d\left({∆h}_{i}\right)$$ (1)

**FIGURE 3:**
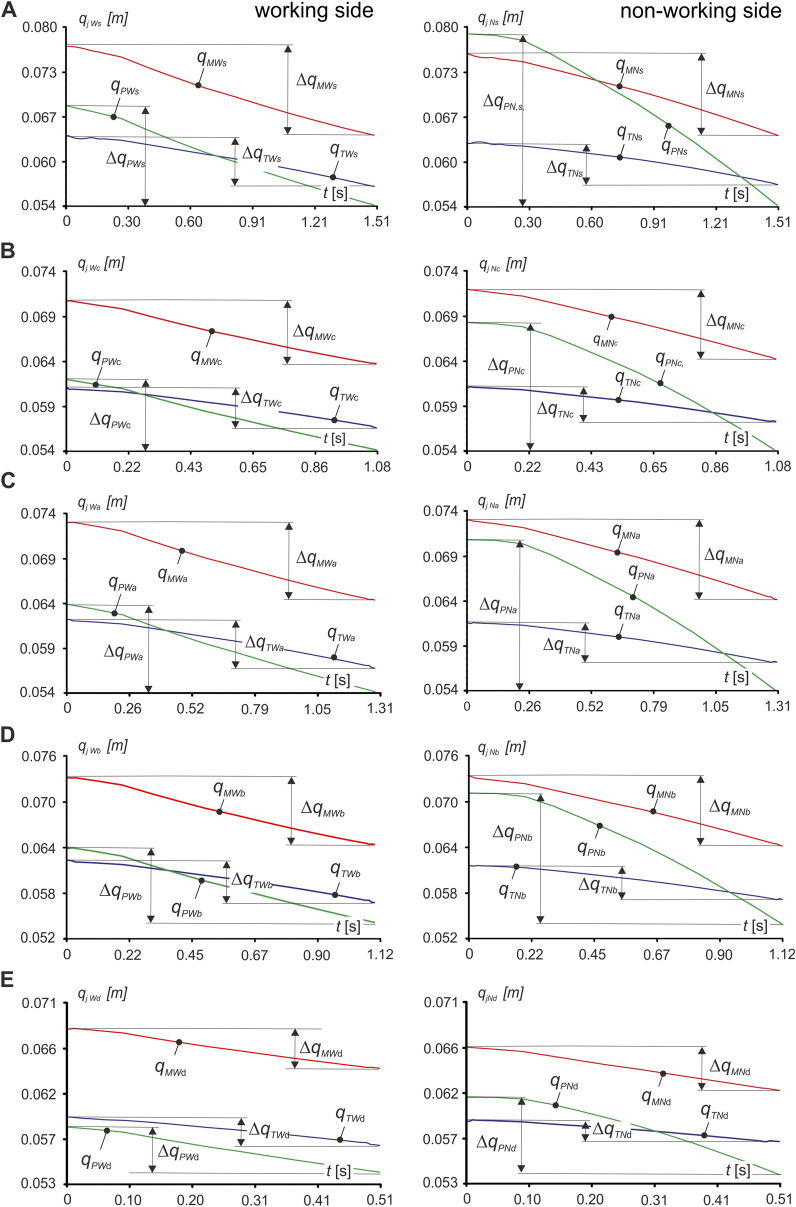
Runs of functions describing the change in muscle length *q*_*jki*_ lying on the right side with molars: on the working side (*W*) and on the non-working side (*N*) when unilateral chewing food: **(A)** sausage *s*, **(B)** carrot *c*, **(C)** apple *a*, **(D)** chocolate bar *b*, **(E)** dark chocolate *d.* (*j* = *M*, *P*, *T*; *k* = *N*, *W*; *i* = *c*, *s*, *a*, *b*, *d*).

Based on the data obtained, after numerical simulations, the characteristics were first determined in the form of a function *q*_*jki*_ = *q*_*jki*_(*t*_*ji*_) i.e., muscle length (*q*_*jki*_) vs time (*t*_*ji*_) (Figure 3), and muscle contraction *Δq*_*jki*_ (Equation 2), both for the working side (*W*) and non-working side (*N*), for the masseter muscle, medial pterygoid muscle and temporalis muscle, respectively.

$$\Delta q_{jki}=\left|q_{jki}\left(t_{ji}\right)\;–\;q_{jki}\left(0\right)\right|$$ (2)

where:

*q*_*jki*_ (0)—initial muscle length for time *t* = 0s-open mouth,

*q*_*jki*_ (*t*_*ji*_)—muscle length determined for time (*t*_*ji*_) from the interval 0÷*t*,

j = M, P, T,

*k* = *W*, *N.*

The contraction velocity *V*_*jkmax.i*_ corresponding to the maximum muscle force *F*_*jkmax.i*_ was determined from Equation 3, for both the working side (*W*) and non-working side (*N*), for the masseter muscle, medial pterygoid muscle and temporalis muscle, respectively.

$$V_{jkmax.i}=\Delta q_{jkmax.i}/t_{jmax.i}$$ (3)

where:

*Δq*_*jkmax.i*_ - muscle contraction corresponding to maximum muscle force for time *t*_*j,max.,i*_, *t*_*jmax.i*_ - contraction time corresponding to maximum muscle force,

*j* = *M, P, T*,

*k* = *W*, *N.*

**FIGURE 4:**
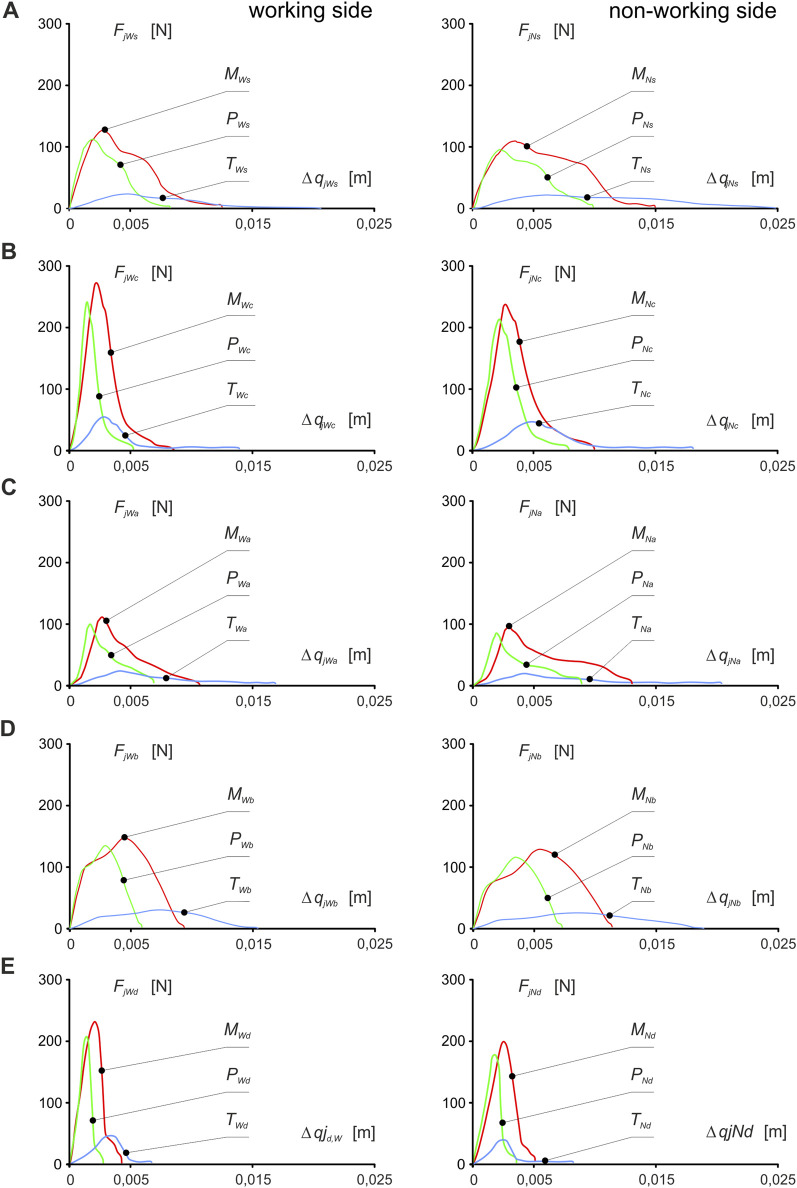
Dynamic patterns of mandibular elevator muscle forces (muscle force vs muscle contraction) about food: **(A)** sausage s, **(B)** carrot c, **(C)** apple a, **(D)** chocolate bar b, **(E)** dark chocolate d and the side of the mandible, i.e. working *(W*) and non-working (*N*) sides, respectively, for the masseter muscle (*FMWi *vs *ΔqMWi, FMNi vs ΔqMNi*), medial pterygoid muscle (*FPWi* vs *ΔqPWi*, *FPNi* vs. *ΔqPNi*) and temporalis muscle (*F_TWi_ vs Δq_TWi_, F_TNi_ vs Δq_TNi_*). . (*j* = *M, P*, *T; i* = *c, s, a, b, d*) - [Stróżyk and Balchanowski, 2023].

Based on the data above, dynamic muscle patterns (muscle force *F*_*jk,max.i*_ vs. muscle contraction *Δq*_*jki*_) were developed (Figure 4) from which the energy values *E*_*jki*_ from Equation 4 and the peak power value *PP*_*jki*_ from Equation 5 generated by a single muscle, after working side and non-working side were calculated (Table 1).

$$E_{jki}=\int_{0}^{{∆q}_{jki}}F_{jki\;}d\left(∆q_{jki}\right)$$ (4)

$$PP_{jki}=F_{jkmax.i}\times V_{jkmax.i}$$ (5)

Where:

**TABLE 1:** Kinematic-dynamic parameters (energy *E*_*jk*_, contraction velocity *V*_*jki*_ corresponding to maximum unilateral chewing force, maximum muscle force *F*_*jkmax.i*_ and peak power *PP*_*jki*_ of the dynamic patterns of elevator muscles of mandible forces (muscle force vs muscle contraction) about food and mandibular side, i.e. working side (*W*) and non-working side (*N*), respectively, for the masseter (masseter muscle (*M*), medial pterygoid muscle (*P*), temporalis muscle (*T*)) depending on food (*i*) and sides of the mandible i.e. working side (*W*) and non-working side (*N*) (*j* = *M*, *P*, *T*; *k* = *W*, *N*) - *The maximum values of muscle forces* (*F*_*jkmax.i*_) *were determined based on the results presented in* Stróżyk and Balchanowski (2023).

Side	Kinematic and dynamic parameters	Sausage (*s*)	Carrot (*c*)	Apple (*a*)	Chocolate bar (*b*)	Chocolate (*d*)
Masetter *(M)*						
Working	*E*_*MWi*_ [J]	0.62	0.49	0.33	0.56	0.18
	*PP*_*MWi*_ [W]	1.90	2.80	0.40	0.95	0.73
	*F*_*MWmax.i*_ [N]	126.9	274.3	112.2	233.2	149.4
	*V*_*MWi*_ [m/s]	0.0162	0.0139	0.0049	0.0091	0.0053
Non-working	*E*_*MNi*_ [J]	0.45	0.36	0.24	0.41	0.13
	*PP*_*MNi*_ [W]	0.85	1.82	0.28	0.78	0.64
	*F*_*MNmax.i*_ [N]	108.8	235.5	96.2	200.3	127.8
	*V*_*MNi*_ [m/s]	0.0116	0.0118	0.0049	0.0088	0.005
Medial pterygoid *(P)*						
Working	*E*_*PWi*_ [J]	0.35	0.24	0.17	0.27	0.08
	*PP*_*PWi*_ [W]	0.81	1.55	0.19	0.49	0.28
	*F*_*PWmax.i*_ [N]	113.5	244.7	100.6	209.3	134.5
	*V*_*PWi*_ [m/s]	0.0074	0.0101	0.0026	0.0053	0.0023
Non-working	*E*_*PNi*_[J]	0.26	0.18	0.13	0.20	0.15
	*PP*_*PNi*_ [W]	0.35	1.42	0.11	0.35	0.25
	*F*_*PNmax.i*_ [N]	97.3	209.8	86.2	179.2	115.4
	*V*_*PNi*_ [m/s]	0.0052	0.0084	0.0022	0.0045	0.0022
Temporalis *(T)*						
Working	*E*_*TWi*_ [J]	0.14	0.11	0.07	0.12	0.04
	*PP*_*TWi*_ [W]	0.41	0.40	0.09	0.20	0.15
	*F*_*TWmax.i*_ [N]	24.4	53.4	22.7	46.1	29.3
	*V*_*TWi*_ [m/s]	0.0176	0.01	0.0053	0.0099	0.0056
Non-working	*E*_*TNi*_ [J]	0.18	0.14	0.09	0.15	0.05
	*PP*_*TNi*_ [W]	0.39	0.44	0.08	0.32	0.14
	*F*_*TNmax.i*_ [N]	20.9	45.9	19.4	39.4	25.0
	*V*_*TNi*_ [m/s]	0.0254	0.0141	0.0074	0.0181	0.0057

*j = M, P, T*,

*k = W, N*.

## 4 Discussion

The analysis of the results obtained from numerical simulations demonstrated that work (energy) and peak power are parameters that can be utilized for qualitative and quantitative assessment of the effect of food (characterized by the function *F*_*i*_ *= f*(*Δh*_*i*_)-force (*F*_*i*_) vs. displacement (*Δh*_*i*_)) on the functioning of the masticatory system during unilateral chewing.

Detailed analysis also revealed that obtaining the same energy value for two foods with differing textures is possible. The results showed no explicit relationship between muscle force and muscle energy, such as a high force value = high energy value. *For example*, In the study (Stróżyk and Bałchanowski, 2023), the maximum muscle forces during unilateral chewing were reported, demonstrating that the masseter muscle generates the most significant force, e.g., for sausage 126.9N and 108.8N and chocolate 233.2N and 200.3N, for the working and non-working sides respectively. In Table 1, the energy values for the masseter muscle for sausage and chocolate were 0.62J and 0.18J for the working side and 0.45J and 0.13J for the non-working side, respectively. The obtained energy values are attributed to differences in the height of sausage and chocolate-the difference was 18.1 mm. This finding indicates that the height of the food significantly affects the contraction of the elevator muscles of the mandible and their energy. A similar relationship to energy was also observed for the muscle’s peak power (Table 1).

The results were analyzed using the percentage difference (|*Δ*|) between values A and B, determined based on the general Equation 6.

$$\left|\Delta \right|=2\left(A‐B\right)\;/\;\left(A+B\right)\times 100\%$$ (6)

### 4.1 Limitations of the model

The model’s limitations include two factors (1): the measurement setup used in experimental studies to determine food characteristics (patterns) in the form of the function *F*_*i*_ *= f*(*Δh*_*i*_), and (2) the numerical model. In the experimental studies, a unilateral chewing simulator replicates a section of the mandibular and maxillary dental arch (Figure 1). The measurement setup allowed rotation about the hinge axis but not the vertical axis. Due to these limitations, food characteristics were determined during the crushing phase for the first cycle of unilateral chewing.

Another limitation is the numerical model of the masticatory system (Figure 2), which included only the elevator muscles of the mandible (masseter, medial pterygoid, and temporalis muscles) (Stróżyk and Bałchanowski, 2016, Stróżyk and Bałchanowski, 2018, Stróżyk and Bałchanowski, 2023; Pachnicz and Stróżyk, 2021) modelled as single vectors (Kashi et al., 2010; Tuijt et al., 2010; Pinheiro et al., 2021). Only one geometric model of the masticatory system was used in the numerical simulations.

The temporomandibular joint was modelled as a contact kinematic pair with five degrees of freedom (three rotations and two displacements) without considering the soft tissues (joint capsule, ligaments and articular disc). In contrast, the connection between the tooth and the body of the mandible did not consider the periodontal ligament with its receptors.

Despite these limitations, the model satisfies the fundamental criteria of solid body mechanics and conditions corresponding to unilateral chewing [boundary conditions (Stróżyk and Bałchanowski, 2018; Stróżyk and Bałchanowski, 2023)]. Thus, it can be used in numerical simulations to identify differences in the functioning of the elevator muscles of the mandible depending on the food texture during unilateral chewing.

### 4.2 Energy of a single muscle

The analysis of the data in Table 1 shows that during unilateral chewing, the masseter muscle (*M*) has the most significant influence on the energy (work) of the muscular system (elevator muscles of the mandible), while the temporalis muscle (*T*) has the least influence, regardless of food type or mandibular side. The average percentage difference between them is *Δ*_*MTW*_ = 127.9% for the working side (*W*) and *Δ*_*MTN*_ = 89.3% for the non-working side (*N*). However, the average percentage difference (regardless of food type) between the medial pterygoid muscle (*P*) and the masseter (*M*) and temporalis (*T*) muscles for the working and non-working sides is *Δ*_*PMW*_ = 67.0% and *Δ*_*PTW*_ = 77.4 and *Δ*_*PMN*_ = 64.4% and *Δ*_*PTN*_ = 28.9%, respectively.

As expected, the results indicate that the masseter and medial pterygoid muscles generate more incredible energy (work) on the working side than on the non-working side. The average percentage difference between sides is *Δ*_*MWN*_ = 31.8% for the masseter muscle and *Δ*_*PWN*_ = 29.5% for the medial pterygoid muscle. In contrast, the temporalis muscle generates more energy on the non-working side than on the working side, which is the opposite of the behaviour observed for the masseter and medial pterygoid muscles. Regardless of food texture, the average difference between sides is *Δ*_*TWN*_ = 25.0%.

These relationships can be interpreted in terms of solid body mechanics, considering the numerical model of the masticatory system (Figure 2C) and the boundary conditions. During unilateral chewing, the masseter and medial pterygoid muscles on the working side overcome the vertical resistance associated with crushing the food. The temporalis muscle on the working side stabilizes and assists (*via* its anterior portion) the masseter and medial pterygoid muscles in pressing the mandibular teeth against the maxillary teeth. This stabilization is due to the temporalis muscle’s role in maintaining the temporary centre of mandibular rotation in the *TMJ* during elevation (Figure 2D). On the non-working side, the masseter and medial pterygoid muscles stabilize the mandible, preventing rotation around the sagittal axis during elevation. Meanwhile, the temporalis muscle retracts the mandible and rotates it about the vertical axis due to the oblique positioning of the mandible relative to the maxilla. Consequently, the temporalis muscle performs work associated with mandibular displacement and rotation, as well as overcoming horizontal resistance of the food linked to the imposed trajectory of the lower incisors (Figure 2D).

### 4.3 Energy of the muscular system

#### 4.3.1 Unilateral chewing

The analysis of total energy (*E*_*TUi*_) determined from numerical simulations (Table 2) indicates that during unilateral chewing, the elevator muscles of the mandible generate the highest energy for sausage (*s*) and the lowest for chocolate (*d*), with respective values of 2.00J and 0.54J. The percentage difference (*Δ*_*ii*_) between the above foods is as high as *Δ*_*sd*_ = 115%. For other foods (Table 2), including a chocolate bar (*b*), apple (*a*), and carrot (*c*), the percentage differences relative to sausage are significantly more minor, amounting to *Δ*_*sb*_ = 16%, *Δ*_*sa*_ = 64%, and *Δ*_*sc*_ = 27%, respectively.

**TABLE 2:** Total energy values obtained from numerical simulations (*E*_*TUi*_) and experimental studies (*E*_*TXi*_), and the percentage differences (*ΔE*_*UXi*_) between them for selected foods (*i*).

Foodstaff (*i*)^a^	Total energy [J]		Percentage difference [%]
	Numerical simulation (*U*)	Experimental studies (*X*)	
	*E*_*TUi*_^b^	*E*_*TXi*_	*ΔE*_*UXi*_
Sausage (*s*)	2.00	1.93	3.6
Carrot (*c*)	1.52	1.48	2.7
Apple (*a*)	1.03	0.98	5.0
Chocolate bar (*b*)	1.71	1.74	1.7
Chocolate (*d*)	0.54	0.53	1.9

^a^ *i = s, c, a, b and d*.

^b^ *E*_*TUi*_ = *E*_*MWi*_ + *E*_*PWi*_ + *E*_*TWi*_+ *E*_*MNi*_ + *E*_*PNi*_ + *E*_*TNi*_ (Table 1).

Table 2 also presents the energy values required to destroy the food samples, determined through experimental studies (Stróżyk and Bałchanowski, 2023). A comparative analysis of the energy for selected products (*i*) shows that the percentage difference (*ΔΕ*_*UXi*_) between the experimental and numerical results is *ΔΕ*_*UXi*_ ≤ 5.0%. The highest percentage difference was observed for the apple *ΔΕ*_*UXa*_ = 5.0%, while the lowest was for the chocolate bar *ΔΕ*_*UXb*_ = 1.7%. The percentage differences for sausage, carrot, and chocolate were *ΔΕ*_*UXs*_ = 3.6%, *ΔΕ*_*UXc*_ = 2.7%, and *ΔΕ*_*UXd*_ = 1.9%., respectively. These results (*ΔΕ*_*UXi*_) indicate good agreement between experimental studies and numerical calculations. The differences are primarily attributed to the limitations of the simulator used in the experimental studies and the constraints of the numerical model.

#### 4.3.2 Unilateral chewing vs symmetrical incisal biting

Based on the energy values (Table 3), it can be observed that on the working side, the highest percentage differences (*ΔΕ*_*WUIi*_) between unilateral chewing and symmetrical incisal biting were noted for the chocolate bar (*ΔΕ*_*WUIb*_ = 119.3%), and the smallest for carrot (*ΔΕ*_*WUIc*_ = 40.0%). In contrast, for sausage and apple, the values are comparable and amount to *ΔΕ*_*WUIs*_ = 102.0% and *ΔΕ*_*WUIa*_ = 96.1%, respectively. For chocolate, the percentage difference value (*ΔΕ*_*WUId*_ = 44.9%) is almost identical to the value obtained for carrot (*ΔΕ*_*WUIc*_ = 40.0%). On the non-working side, the percentage differences are lower than on the working side, with an average of 16% for sausage, apple, and chocolate bars and 50% for carrots and chocolate.

**TABLE 3:** Percentage differences between: (1) energy determined for unilateral chewing (*E*_*WUi*_; *E*_*WIi*_) and symmetrical incisal biting (*E*_*NUi*_, *E*_*NIi*_), for the working side (*ΔE*_*WUIi*_) and non-working side (*ΔE*_*NUIi*_) and (2) total energy (*ΔE*_*UIi*_) for unilateral chewing (*E*_*TUi*_) and symmetrical incisal biting (*E*_*TIi*_), depending on the food type (*i*).

Working side (*W*)			Non-working side (*N*)			Total Energy [J]		Percentage difference [%]
Energy [J]		Percentage difference [%]	Energy [J]		Percentage difference [%]			
Unilateral chewing	Symmetric incisal biting		Unilateral chewing	Symmetric incisal biting				
*E*_*WUi*_ ^b^	*E*_*WIi*_ ^c^	*ΔE*_*WUIi*_	*E*_*NUi*_ ^b^	*E*_*WIi*_ ^c^	*ΔE*_*NUIi*_	*E*_*TUi*_ ^d^	*E*_*TIi*_e	*ΔE*_*UIi*_
Sausage *(s)*								
1.11	0.36	102.0	0.89	0.36	84.8	2.00	0.72	94.1
Carrot *(c)*								
0.84	0.56	40.0	0.68	0.56	19.41	1.52	1.12	30.3
Apple *(a)*								
0.57	0.20	96.1	0.46	0.20	78.8	1.03	0.40	88.1
Chocolate bar *(b)*								
0.95	0.24	119.3	0.76	0.24	104.0	1.71	0.48	112.3
Chocolate *(d)*								
0.30	0.19	44.9	0.24	0.19	23.33	0.54	0.38	34.8

^a^ *i* = *s, c, a, b* and *d*.

^b^ *E*_*WUi*_ = *E*_*MWi*_+ *E*_*PWi*_+ *E*_*TWi*_ and *E*_*NUi*_= *E*_*MNi*_+ *E*_*PNi*_+ *E*_*TNi*_ (Table 1).

^c^ Stróżyk and Bałchanowski (2020).

^d^ *E*_*TUi*_ = *E*_*WUi*_ + *E*_*NUi*_.

^e^ *E*_*TIi*_ *= E*_*WIi*_ *+ E*_*NIi*_.

The results of the total energy of the masticatory system (*E*_*TUi*_) for unilateral chewing (Table 2) enable comparison with those for symmetrical incisal biting (Stróżyk and Bałchanowski, 2020). For a detailed comparative analysis, it was first established that the energy (*E*_*TUi*_) can be divided in a 55.4/44.6% ratio (Table 2) between the working side (*E*_*WUi*_) and the non-working side (*E*_*NUi*_) (Table 3). Meanwhile, the total energy of symmetrical incisal biting (*E*_*TIi*_), as per the assumptions in Stróżyk and Bałchanowski (2020), is divided equally (50.0/50.0%) between the working side (*E*_*WIi*_) and the non-working side (*E*_*NIi*_) (Table 3).

An analysis of the percentage differences (*ΔΕ*_*UIi*_) between the total energy of unilateral chewing (*E*_*TUi*_) and symmetrical incisal biting (*E*_*TIi*_) indicates that the highest percentage differences are observed for the chocolate bar (*ΔΕ*_*UIb*_112.3), sausage (*ΔΕ*_*UIs*_ = 94.1%) and apple (*ΔΕ*_*UIa*_ = 88.1%), while the most minor differences are for chocolate (*ΔΕ*_*UId*_ = 34.8%) and carrot (*ΔΕ*_*UIc*_ = 30.3%) (Table 3).

The results presented in Table 3 demonstrate that energy differences primarily depend on (1) the food texture and (2) the food’s position on the dental arch, i.e., the mechanisms of food damage.

Based on solid mechanics, it can be shown that the internal structure of materials significantly influences their mechanical parameters. Similar relationships can be observed when analyzing the biting and/or chewing of foods with different textures. Results reported in the literature (Agrawal et al., 1998; Fitts et al., 1991; Hiiemae et al., 1996; Koolstra, 2002; Mathevon et al., 1995; Mioche et al., 1999; Shimada et al., 2012; Stróżyk and Bałchanowski, 2018; Stróżyk and Bałchanowski, 2023), indicate that the mechanical properties of food have a significant effect on muscle activity patterns and muscle force values during the act of chewing. A second important parameter that significantly impacts muscle characteristics will be the position of the food on the dental arch, on which the opening of the mouth - and thus the contraction of the muscle - depends at a fixed food height.

For example, by analyzing a particular food during symmetrical incisor biting and unilateral chewing, assuming a fixed height, it can be shown that during symmetrical incisor biting, the mouth opening is less than during chewing, i.e., the muscles will have different initial lengths, resulting in different contractions. Furthermore, as mentioned, the position of the food also forces the activation of other mechanisms of food damage.

When comparing symmetrical incisal biting and unilateral chewing, it becomes apparent that these are two distinct processes in terms of solid mechanics and deformable body mechanics. Specifically, symmetrical incisal biting involves a single cycle in which the incisors cut the food into two parts and transport it to the molars for substantial dimensional reduction and concurrent modification of their internal structure (texture). The mandibular movements primarily occur in the sagittal plane, with slight lateral deviations in the frontal plane. Consequently, the support and loading conditions are quasi-symmetrical. Therefore, it can be assumed that during mandibular movement, the condyle trajectories, left and right, are identical (Stróżyk and Bałchanowski, 2016). Furthermore, the position of the food bite on the incisors, its loading along the line, and the wedge-shaped structure of the incisors indicate that the biting process is analogous to typical shearing.

Unlike symmetrical incisal biting, unilateral chewing is a multi-cycle process where the number of cycles depends on the changing mechanical parameters, food texture, and individual characteristics. The food damage process primarily involves crushing and grinding food between surfaces formed by two pairs of corresponding molars or one pair of premolars and one pair of molars, depending on individual characteristics.

The above shows that the damage process (food positioning on the dental arch) significantly affects bite force, mouth opening, and muscle contraction. Considering the information above, it is evident that during chewing, muscle energy and/or the energy of the masticatory system depends on the food stiffness.

### 4.4 Peak power of the elevator muscles of the mandible

#### 4.4.1 Unilateral chewing

An analysis of peak power values for the mandibular elevator muscles indicates that the masseter muscle generates the highest power during unilateral chewing. In contrast, the temporalis muscle generates the lowest, regardless of the side of the mandible and the food type (Table 1). The average percentage difference between the masseter and temporalis muscles for the working and non-working sides amounts to 134.1% and 104.9%, respectively. The medial pterygoid muscle generates less power than the masseter but more than the temporalis muscle (Table 1). The average percentage difference between the medial pterygoid and the masseter and temporalis muscles for the working side is 69.6% and 82.4%, respectively, and for the non-working side, it is 75.2% and 36.8%.

Analysis of peak power values in relationship to the mandibular side indicates that the elevator muscles of the mandible generate greater power on the working side than on the non-working side (Table 4). Calculations indicate that the highest percentage difference between the sides (*ΔPP*_*WNUi*_) was observed for sausage (*ΔPP*_*WNUs*_ = 36.5%), while the smallest was for the chocolate bar (*ΔPP*_*WNUb*_ = 11.9%). The carrot, chocolate, and apple values are *ΔPP*_*WNUc*_ = 27.1%, *ΔPP*_*WNUd*_ = 19.8%, and *ΔPP*_*WNUa*_ = 14.6%, respectively.

**TABLE 4:** Peak power for the working side (*PP*_*WUi*_) and non-working side (*PP*_*NUi*_), percentage differences (*ΔPP*_*WNUi*_) between them, and total peak power (*PP*_*TUi*_) of the masticatory system, depending on the food type (*i*).

Foodstaff^a^	Power (*PP*) [W]			
	Working side (*W*)	Non-working side (*N*)	Percentage difference [%]	Total power
	*PP*_*WUi*_ ^b^	*PP*_*NUi*_ ^c^	*ΔPP*_*WNUi*_	*PP*_*TUi*_ ^d^
Sausage (*s*)	3.33	2.30	36.5	5.63
Carrot (*c*)	6.82	5.19	27.1	12.01
Apple (*a*)	0.93	0.80	14.6	1.73
Chocolate bar (*b*)	2.36	2.10	11.9	4.46
Chocolate (*d*)	1.98	1.62	19.2	3.60

^a^ *i* = *s, c, a, b*, and *d*.

^b^ *PP*_*WUi*_ = *PP*_*MWUi*_ + *PP*_*PWUi*_ + *PP*_*TWUi*_ (Table 1).

^c^ *PP*_*NUi*_ = *PP*_*MNUi*_ + *PP*_*PNUi*_ + *PP*_*TNUi*_ (Table 1).

^d^ *PP*_*TUi*_ = *PP*_*WUi*_ + *PP*_*NUi*_.

Analyzing the masticatory system in terms of total peak power (*PP*_*TUi*_), it was found that the elevator muscles of the mandible must generate the highest power for carrot (*PP*_*TUc*_ = 12.01W) and the lowest for apple (*PP*_*TUa*_ = 1.73W) (Table 4). For the remaining foods, the total peak power is ranked as follows: sausage (*PP*_*TUs*_ = 5.63W), chocolate bar (*PP*_*TUb*_ = 4.46W), and chocolate (*PP*_*TUd*_ = 3.60W). Interestingly, three foods (carrot, chocolate bar, and apple) of similar heights (Figure 1) require the muscular system to generate different power values (Table 4). The results indicate that food texture significantly affects the power generated by the muscular system, which depends on the muscular force and contraction velocity of individual foods (Table 1).

Additionally, the masseter and medial pterygoid muscles generate higher peak power on the working side than on the non-working side (Table 1). In contrast, the temporalis muscle generates higher peak power on the non-working side than on the working side. This results from the fact that the muscle must achieve a more significant contraction (Stróżyk and Bałchanowski, 2023) on the non-working side than on the working side. Consequently, to meet the demands of a simple mechanical model (e.g., simultaneous increase in contraction and muscle force values and reaching their maxima at the same time), the muscles must contract at different velocities, i.e., the contraction velocity on the non-working side must be higher than on the working side (Table 1). As a result, despite lower maximum force values on the non-working side, the temporalis muscle generates higher peak power.

#### 4.4.2 Unilateral chewing vs. symmetrical incisal biting

The peak power values (Table 5) show that percentage differences can be divided into two groups, regardless of the mandibular side, i.e., above and below 100%. The first group includes carrots, sausages, and chocolate bars, while the second group comprises apples and chocolate.

**TABLE 5:** Percentage differences between (1) peak power values obtained for unilateral chewing (*PP*_*WUi*_ *, PP*_*NUi*_) and symmetrical incisal biting (*PP*_*Wii*_ *, PP*_*NIi*_), on the working side (*ΔPP*_*WUIi*_) and non-working side (*ΔPP*_*NUIi*_) and (2) total peak power (*ΔPP*_*UIi*_) obtained for unilateral chewing (*PP*_*TUi*_) and symmetrical incisal biting (*PP*_*TIi*_), depending on the food type (*i*).

Working side (*W*)			Non-working side (*N*)					Total Power [W]		Percentage difference [%]
Power [W]		Percentage difference [%]	Power [W]				Percentage difference [%]			
Unilateral chewing	Symmetric incisal biting		Unilateral chewing		Symmetric incisal biting					
*PP*_*WUi*_ ^b^	*PP*_*WIi*_ ^c^	*ΔPP*_*WUIi*_	*PP*_*NUi*_ ^b^		*PP*_*WIi*_ ^c^		*ΔPP*_*NUIi*_	*PP*_*TUi*_ ^d^	*PP*_*TIi*_ ^e^	*ΔPP*_*UIi*_
Sausage *(s)*										
3.33	0.72	128.8	2.30	0.72		104.6		5.62	1.44	118.5
Carrot *(c)*										
6.82	1.39	132.3	5.19	1.39		115.5		12.01	2.78	124.8
Apple *(a)*										
0.93	0.44	71.7	0.80	0.44		58.6		1.74	0.88	65.5
Chocolate bar *(b)*										
2.36	0.60	119.0	2.10	0.60		111.0		4.46	1.20	115.2
Chocolate *(d)*										
1.98	1.05	61.2	1.62	1.05		42.7		3.60	2.10	52.5

^a^ *i* = *s, c, a, b* and *d*.

^b^ (Table 1).

^c^ The authors’ own unpublished data for this paper.

^d^ *PP*_*TUi*_ = *PP*_*WUi*_ + *PP*_*NUi*_.

^e^ *PP*_*TIi*_ = *PP*_*WIi*_ + *PP*_*NIi*_.

The percentage differences between unilateral biting and biting in the first group indicate that the highest value for the working side is observed for carrot, followed by sausage and the lowest value for chocolate bar *ΔPP*_*WUIc*_ = 132.3%, *ΔPP*_*WUIs*_ = 128.8% and *ΔPP*_*WUIb*_ = 119.0%, respectively, while for the non-working side the highest values are also for carrot (*ΔPP*_*NUIc*_ = 115.5%), and the lowest for sausage (*ΔPP*_*NUIs*_ = 104.6%). For chocolate bars, the percentage difference value is *ΔPP*_*NUIb*_ = 111.0%.

In the second group, the highest value was observed for apple and the lowest for chocolate, respectively, on the working side: *ΔPP*_*WUIa*_ = 71.7% and *ΔPP*_*WUId*_ = 61.2% and on the non-working side: *ΔPP*_*NUIa*_ = 58.6% and *ΔPP*_*NUId*_ = 42.7%.

The results obtained for chocolate are surprising. However, considering (1) its small height relative to other foods (Table 1; Figure 1) and (2) its brittle material properties, the results obtained at this stage of the research should be regarded as highly probable.

Analysis of the percentage differences (*ΔPP*_*UIi*_) between total peak power during unilateral chewing (*PP*_*TUi*_) and symmetrical incisal biting (*PP*_*TIi*_) indicates that the most significant differences occur for carrot (*ΔPP*_*UIc*_ = 124.8%), and the smallest for chocolate (*ΔPP*_*UId*_ = 52.5%). The values for sausage and chocolate bar are almost identical, amounting to *ΔPP*_*UIs*_ = 118.5% and *ΔPP*_*UIb*_ = 115.3%, respectively. For apple, the percentage difference (*ΔPP*_*UIa*_ = 65.5%) is 13% higher than that for chocolate.

Analyzing these results for individual foods reveals that the power of the muscle system is strongly dependent on the food’s location on the dental arch. This dependency is most pronounced for carrot, sausage, and chocolate bar, with an average percentage difference of 119.5%. Differences between apple and chocolate are also apparent but not as significant as for the aforementioned foods - the average difference is 59.0%.

Considering the results in Tables 4 and 5, it can be concluded that differences in muscular system forces depend primarily on (1) the texture of the food and (2) the position of the food on the dental arch, i.e., the mechanism of food destruction (biting and unilateral chewing).

Furthermore, based on the percentage differences between peak power during unilateral chewing and symmetrical incisal biting, the following hypothesis can be proposed: the mechanism of food damage has a significantly more significant impact on the peak power of the muscle system for foods with high texture heterogeneity than for foods with low texture heterogeneity.

## 5 Conclusion

Analysis of the results showed that energy and peak power can be used to quantitatively and qualitatively assess the elevator muscle of the mandible during the first cycle of unilateral chewing of foods with different textures (Stróżyk and Balchanowski, 2023). Furthermore, the study showed that these parameters can also be used to compare two kinematically and dynamically different stages of the chewing act, i.e., incisal biting and unilateral chewing.

Additionally, it should be noted that muscle-generated energy depends on two parameters (1): muscle force and (2) contraction, which in turn depend on bite force (food texture) and food height (mouth opening), respectively. Bite force and mouth opening are influenced by the food’s position on the dental arch. Regarding peak muscle power, the contraction velocity during symmetrical incisal biting and/or unilateral chewing must also be considered. This velocity, in turn, depends on time, food height, position on the dental arch, food texture, and individual characteristics.

A method to determine which of these parameters significantly affects energy and peak power patterns is dimensional analysis (Buckingham’s Pi theorem) (Gibbings, 2011).

Since experimental studies and numerical simulations were carried out for only five food products (Figure 1), the results and conclusions must be verified for products with different internal textures and heights.

In addition, a complete understanding of the function of the masticatory system during the act of chewing requires *in vivo* studies, which should take into account the effects of age, dental status, masticatory dysfunction and dietary habits (type of diet) on the velocity of chewing, and thus on the contraction velocity of the mandibular elevator muscles.

The results obtained can be used in (I) clinical practice, e.g., for the evaluation of temporomandibular joint disorders, planning of orthodontic and prosthetic treatment, i.e., design of braces, prostheses and implants of the masticatory system, maxillofacial surgery, i.e., planning of mandibular reconstruction, therapy after fractures and after Bilateral Sagittal Split Osteotomy-BSSO), (II) rehabilitation, e.g.,: development of exercise programs for patients after injury, surgery and those suffering from muscle weakness, optimization of therapy for bruxism, development of muscle monitoring devices, (III) bionic systems of the masticatory system, e.g.,: design of mandibular and maxillary prostheses taking into account the natural loads and functioning of the muscular system, development of robots to assist in masticatory therapy and rehabilitation of the masticatory system after injuries, development of nerve impulse-controlled muscle implants that mimic their natural functions, (IV) biomechanical research, e.g., primarily, development of numerical dynamic models of the masticatory system based on finite element methods-FEM and carrying out simulations and analyses for selected boundary conditions - muscle characteristics, position of the mandible.

## Data Availability

The original contributions presented in the study are included in the article/Supplementary Material, further inquiries can be directed to the corresponding author.
